# Predictive and Prognostic Importance of National Early Warning Score 2 (NEWS2) in Emergency Room Patients

**DOI:** 10.7759/cureus.79538

**Published:** 2025-02-24

**Authors:** Abin Thomas, Naveen Mohan, Jerin Varghese, Gireesh Kumar, Sreekrishnan Trikkur, Sabarish B Nair, Bharath Prasad S, Ritvik Sajan, Manna M Theresa

**Affiliations:** 1 Department of Emergency Medicine, Amrita Institute of Medical Sciences and Research, Kochi, IND; 2 Department of Emergency Medicine, Amrita Institute Of Medical Sciences and Research, Kochi, IND; 3 Department of Pharmacy Practice, Amrita Institute of Medical Sciences and Research, Kochi, IND

**Keywords:** clinical outcomes, emergency department, mortality rate, news2 (national early warning score 2), patient deterioration, prognostic capabilities, sensitivity and specificity

## Abstract

Introduction

The National Early Warning Score 2 (NEWS2), a modified version of its predecessor NEWS, is a screening tool developed by the Royal College of Physicians and serves as a stratification tool/scale that classifies patients based on six physiological parameters. Despite being widely recognized for its excellent accuracy, there is limited evidence regarding its application within the Indian population and its role in resource allocation to enhance patient care. This study aims to evaluate the predictive accuracy of NEWS2 in determining mortality among Indian patients, aiding in proper risk stratification and improving the quality of care.

Materials and methods

This prospective observational study was conducted over six months in the emergency department of a South Indian tertiary care hospital. Out of 150 patients screened, 101 (n = 101) were included after obtaining informed consent and meeting the inclusion criteria. The study aimed to assess the predictive accuracy of NEWS2 and analyze the distribution of its individual physiological parameters.

Results

The study showed a significant difference in NEWS2 scores between survivors (n = 87, mean 4.36, SD 2.698) and non-survivors (n = 14, mean 13.14, SD 1.406), highlighting its potential as a valuable tool for risk stratification. Of the 101 patients, 86.1% (n = 87) survived and 13.9% (n = 14) died. Survivors (n = 87) had a lower mean NEWS2 score of 4.36 ± 2.698, whereas non-survivors (n = 14) had a significantly higher mean score of 13.14 ± 1.406.

Conclusion

Our findings demonstrate that higher NEWS2 scores at admission are strongly correlated with mortality rates. These results validate NEWS2's effectiveness in identifying patients at risk of deterioration, affirming its role as a critical tool for early intervention in emergency care.

## Introduction

Emergency medicine is a medical specialty where prompt, accurate, and timely assessment of patient conditions is essential for effective management and better outcomes. A crucial aspect of timely patient management is identifying high-risk patients and differentiating them from stable patients. Crilly et al. report that 1.0%-2.4% of emergency department cases require intensive care unit (ICU) admission [1]. This rate may vary globally, especially during events like a novel pandemic, which can significantly increase high-risk patient admissions.

Apart from high admission rates, the unavailability of ICU beds also poses a challenge to timely patient care. Bed overcrowding by non-risk patients, improper risk-based patient classification, delays in triage-to-ICU transfer, bed shortages, and inefficient resource distribution all impact the quality of patient care [2-5]. Mathews et al. found a concerning link between delayed ICU admissions and increased post-hospitalization morbidity [2]. The delay was attributed to the time spent on risk stratification and the lack of available beds.

To ensure timely patient intervention, different risk-based stratification tools/scales are used to guide appropriate healthcare delivery. Tools such as the Revised Trauma Score (RTS), Trauma and Injury Severity Score (TRISS), Acute Physiology and Chronic Health Evaluation (APACHE) II Score, quick Sequential Organ Failure Assessment (qSOFA) score, and Charlson Comorbidity Index (CCI) [6-10] have been developed. One such screening tool, the National Early Warning Score (NEWS), developed by the Royal College of Physicians in 2012, was modified and introduced as NEWS2 in 2017 [11,12]. Unlike its predecessor, NEWS2 improved by adding parameters such as respiratory rate, oxygen saturation, systolic blood pressure, pulse rate, level of consciousness, and body temperature, enhancing its accuracy and responsiveness [11,12]. Studies indicate that NEWS2 is beneficial in the emergency department for patient risk categorization and predicting mortality and morbidity [13-17]. Due to the need for quick decision-making, NEWS2 offers a significant advantage over other risk stratification tools such as RTS and TRISS [10]. Despite its proven accuracy and efficiency, its effectiveness across different populations remains a topic of ongoing investigation.

This prospective observational study aims to validate the effectiveness of NEWS2 screening in the emergency department of a tertiary care center serving a predominantly Indian population. The findings could support the implementation of NEWS2 for patient screening in this population.

## Materials and methods

Study design and setting

This prospective observational study was conducted over six months in the emergency department of Amrita Institute of Medical Sciences, Kochi, Kerala, India. This setting is chosen for its high patient volume and diverse medical cases, which is ideal for evaluating the NEWS2 scoring system across a broad spectrum of emergency presentations. The NEWS2 score was calculated for all patients as part of their initial admission examination. The study was approved by the Institutional Review Board and Ethics Committee of Amrita School of Medicine (ethics number: ECASM-AIMS-2023-466), as no ethical or scientific concerns were involved in this study. Participants were included after providing verbal and signed informed consent.

Study participants

The study population consisted of participants screened according to specific inclusion and exclusion criteria. Inclusion criteria were adult patients (aged 18 and above) presenting to the emergency department, for whom a NEWS2 score was calculated as part of the initial admission assessment. Exclusion criteria included patients on mechanical ventilation upon arrival, those who expired before the NEWS2 score could be recorded, those who did not provide informed consent, those under the influence of alcohol at the time of presentation, pregnant women, patients with primary psychiatric pathology, and those who left against medical advice before data collection was completed. Prior to conducting the main study, a pilot study was conducted with 10 participants to gather initial data for sample size calculation. The sample size was calculated using the formula n = (Zα/2 × σ / E)², where Zα/2 is the Z value for a 95% confidence level (1.96), σ is the standard deviation (3.53), and E is the allowable error (10% of the mean, 7.5). This calculation yielded a minimum required sample size of 86 participants. To account for potential dropouts and to enhance the study's statistical power, the study aimed to recruit a larger sample. Ultimately, 101 participants were enrolled in the study. Of the 150 patients screened, 101 met the inclusion criteria and 49 were excluded from the study. 

Definitions

The NEWS2 score, a modified version of the NEWS score developed by the Royal College of Physicians, takes into account six physiological parameters: respiratory rate, oxygen saturation, body temperature, systolic blood pressure, heart rate, and level of consciousness. Each parameter is assigned points from zero to three, with three indicating the highest severity. An additional two points are added if the patient is on supplemental oxygen. Patients are classified into risk categories based on their total score: low risk (0-4 points), low to medium risk (3 points in any single parameter), medium risk (5-6 points), and high risk (≥7 points) [11,12].

Objectives

Our study primarily aimed to validate the effectiveness of NEWS2 in predicting patient deterioration in emergency settings. This involves assessing whether NEWS2 can reliably identify patients at risk of worsening conditions early enough to allow timely and effective interventions. Additionally, the study seeks to determine the distribution of individual physiological parameters within the study population.

Methodology

**Figure 1:**
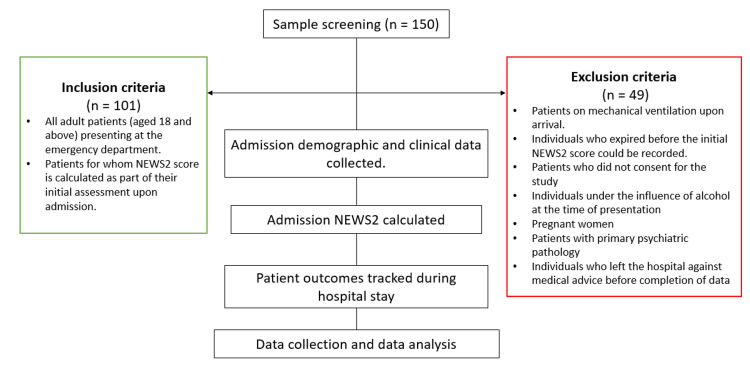
Representation of the study methodology NEWS2: National Early Warning Score 2.

Demographic details of all participants were collected and analyzed, with recruitment based on inclusion and exclusion criteria. All participants provided oral and written informed consent for clinical data collection, which was transcribed into physical and electronic medical records. Data on demographics, comorbidities, and clinical presentation were obtained for each patient. The NEWS2 at hospital admission was calculated using the patient's physiological parameters from medical records. Throughout the patient's hospital stay, patient data were monitored, and the study objectives were analyzed. The study methodology is summarized in Figure 1.

Data collection

Data collection was carried out by a team consisting of the principal investigator, emergency medical technician students, and nurses in the emergency department to ensure comprehensive and timely data collection. A standardized format was used to capture demographic details, individual components of the NEWS2 score, and patient outcomes. Data collection was systematic and adhered to the hospital’s standard protocol for emergency admissions. NEWS2 was recorded at admission and at regular intervals, as per the hospital’s standard operating procedures. Patient outcomes, including hospital admission, length of stay, ICU transfer, and mortality, were tracked. Demographic and clinical data, such as age, sex, diagnosis, and comorbidities, were also collected to control for potential confounders in the analysis.

Statistical analysis

Statistical analyses were conducted using IBM SPSS Statistics for Windows, Version 20.0 (Released 2011; IBM Corp., Armonk, NY, United States). Descriptive statistics were initially used to describe the distribution of NEWS2 scores and patient outcomes, providing a foundational understanding of the data. This included calculating means, standard deviations, and ranges for NEWS2 scores, as well as frequencies for various patient outcomes. An independent t-test was conducted to compare NEWS2 scores between survivors and non-survivors. The significance level was set at p < 0.05. Additionally, sensitivity and specificity calculations were performed at various NEWS2 score thresholds to determine optimal cutoff points. A component analysis of individual NEWS2 parameters was also conducted to examine the distribution and contribution of each physiological measure to the overall score.

## Results

The descriptive statistics for the 101 patients (n = 101) are provided in Table 1. The mean age of male participants (n = 50) was 52.90 years (SD ± 22.09), while the mean age of female participants (n = 51) was 52.82 years (SD ± 21.70). No significant age difference was found between the two groups (p = 0.984), as determined by an independent t-test.

**Table 1:** Demographic and clinical characteristics of male and female patients LOC: loss of consciousness, SD: standard deviation.

Characteristic	Male	Female	p-value
Age (years, mean ± SD)	52.90 ± 22.09	52.82 ± 21.70	0.984
NEWS2 score (mean ± SD)	7.25 ± 3.87	8.16 ± 4.35	0.267
LOC = 0 (n%)	55.77%	46.94%	0.491
LOC = 3 (n%)	44.23%	53.06%	0.491

The mean NEWS2 score for male participants (n = 50) was 7.25 (SD ± 3.87), compared to 8.16 (SD ± 4.35) for female participants (n = 51). The difference in NEWS2 scores between genders was not statistically significant (p = 0.267).

Regarding the level of consciousness (LOC), 55.77% of male participants (n = 50) had an LOC score of 0, indicating normal consciousness, compared to 46.94% of female participants (n = 51) with the same score. Conversely, 44.23% of male participants (n = 50) had an LOC score of 3, indicating a reduced level of consciousness, while 53.06% of female participants (n = 51) fell into this same category. The chi-square test showed no statistically significant difference in LOC distribution between male and female participants (p = 0.491).

**Figure 2:**
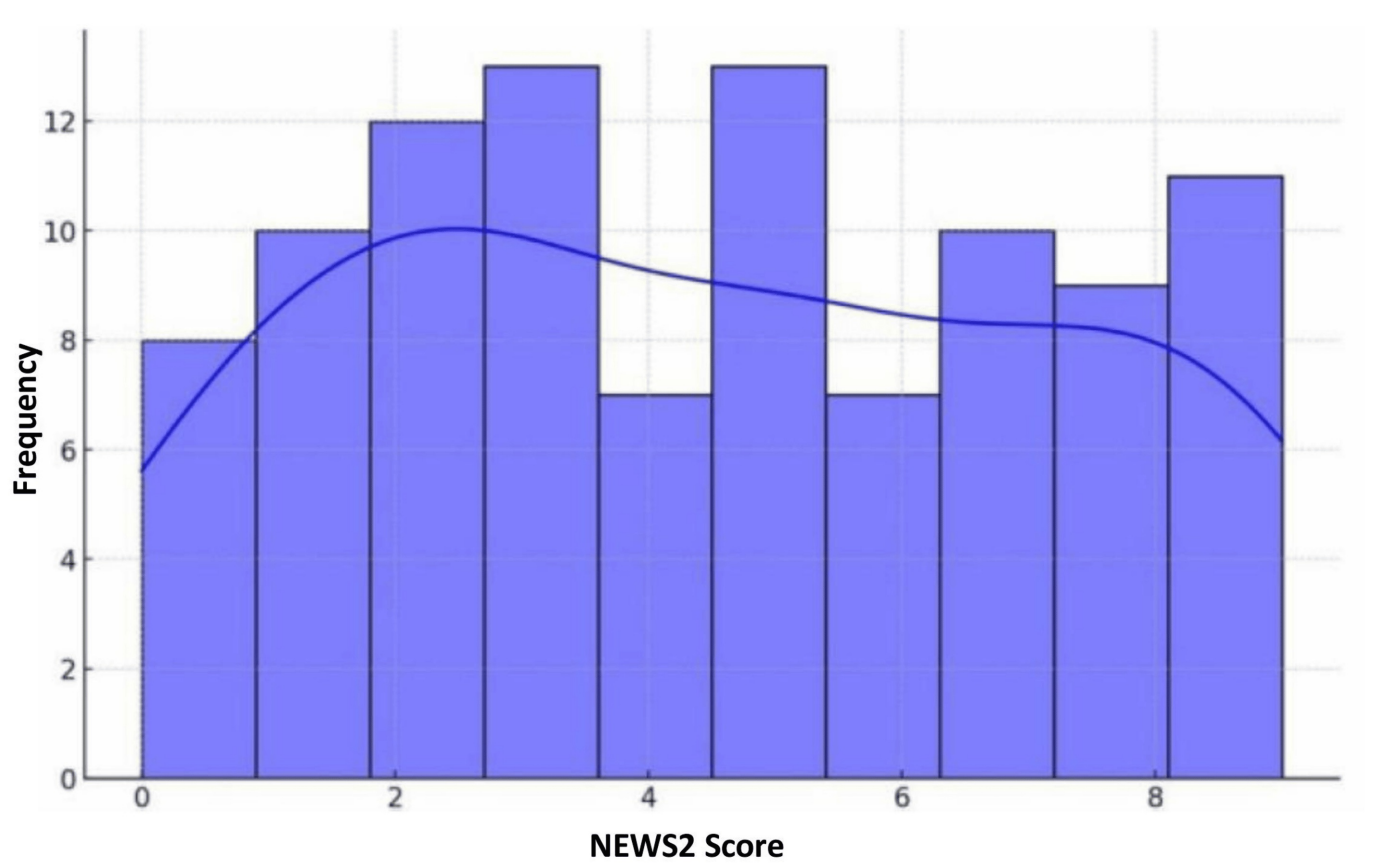
Distribution of NEWS2 score at admission

The distribution of the NEWS2 score at the time of admission of the patients is summarized in Figure 2. 

The study analyzed the predictive and prognostic importance of the NEWS2 score in emergency room patients. For respiratory rate, most patients scored 0 (n = 31, 30.7%) or 1 (n = 33, 32.7%). SpO2 levels were predominantly at score 0 (n = 49, 48.5%) or 2 (n = 42, 41.6%). Systolic blood pressure primarily scored 0 (n = 63, 62.4%), while pulse rate showed a higher distribution at a score of 1 (n = 17, 53.5%). LOC was either 0 (n = 70, 69.3%) or 3 (n = 31, 30.7%). Temperature had the highest distribution at scores of 0 (n = 39, 39%) and 1 (n = 45, 45%). These variations in NEWS2 scores demonstrate its effectiveness in predicting sepsis severity and patient outcomes in the emergency department (Table 2).

**Table 2:** General distribution of individual NEWS2 score parameters SpO2: oxygen saturation.

Variables	NEWS2 aggregate score			
	0	1	2	3
Respiratory rate (n, n%)	31 (30.7%)	33 (32.7%)	15 (14.9%)	22 (21.8%)
SpO2 (n, n%)	49 (48.5%)	10 (9.9%)	42 (41.6%)	n/a
Systolic blood pressure (n, n%)	63 (62.4%)	17 (16.8%)	21 (20.8%)	n/a
Pulse rate (n, n%)	17 (16.8%)	54 (53.5%)	30 (29.7%)	n/a
Level of consciousness (n, n%)	70 (69.3%)	n/a	n/a	31 (30.7%)
Temperature (n, n%)	39 (39%)	45 (45%)	17 (17%)	n/a

**Figure 3:**
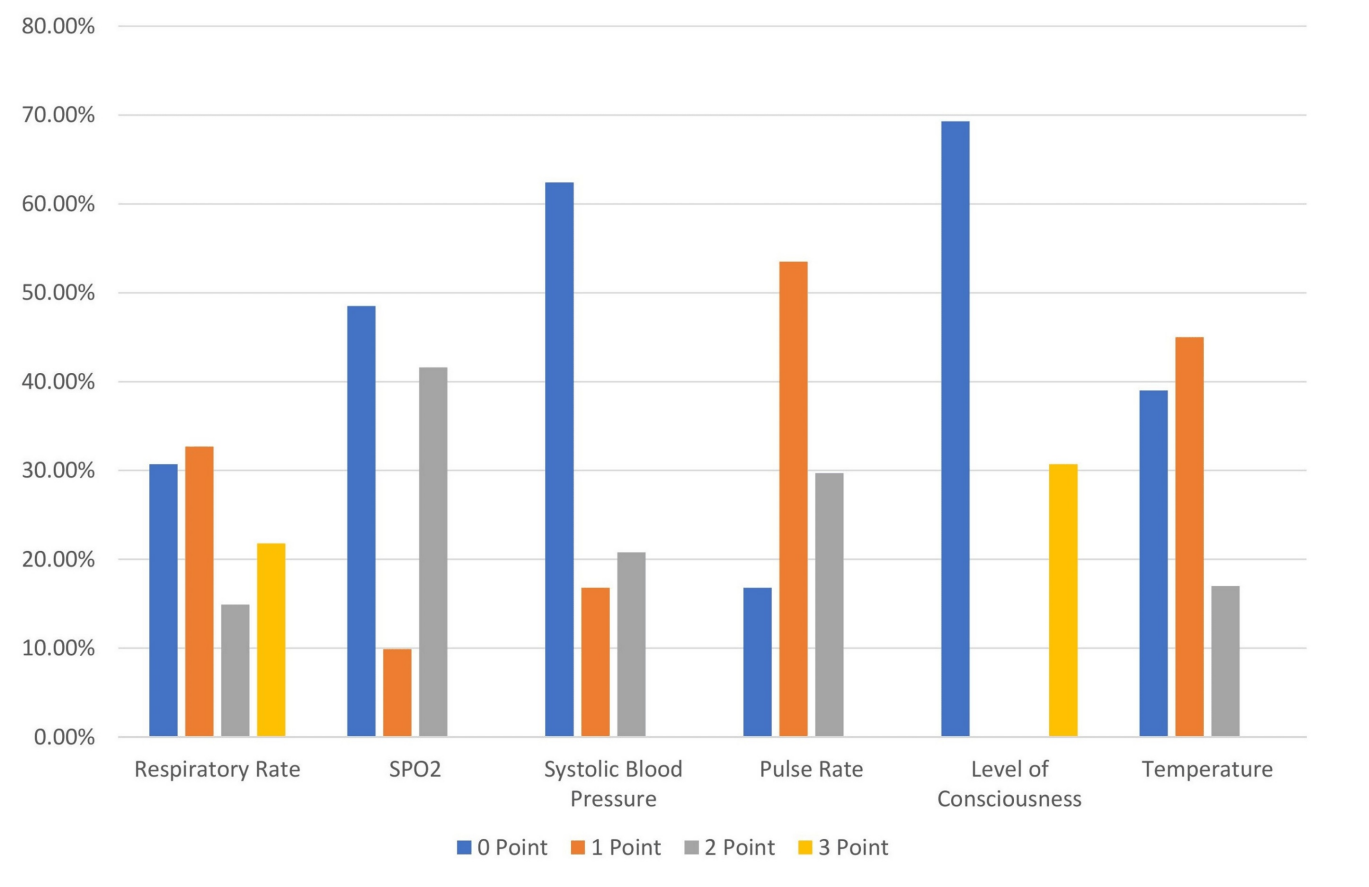
NEWS2 aggregate score SpO2: oxygen saturation.

Figure 3 presents the NEWS2 score distribution graphically and individual distribution for various parameters: respiratory rate had the highest scores at 0 (n = 31, 30.7%) and 1 (n = 33, 32.7%), SpO2 levels were mostly 0 (n = 49, 48.5%) and 2 (n = 42, 41.6%), systolic blood pressure predominantly scored 0 (n = 63, 62.4%), pulse rate was highest at 1 (n = 54, 53.5%), LOC was split between 0 (n = 70, 69.3%) and 3 (n = 31, 30.7%), and temperature had major scores at 0 (n = 39, 39%) and 1 (n = 45, 45%). 

Table 3 shows that the NEWS2 scores for 101 samples (n = 101) had a mean of 5.57 and a standard deviation of 3.98, with scores ranging from 1.00 to 14.00. This variability indicates that the NEWS2 score effectively differentiates between varying levels of clinical severity, with higher scores predicting more severe conditions and potentially worse outcomes. Thus, the NEWS2 score is valuable for assessing patient urgency and guiding treatment decisions.

**Table 3:** Analysis of obtained scores

Parameters	Results
Total participants (n)	101
Mean	5.574
Std. deviation	3.978
Minimum	1.00
Maximum	14.00

Table 4 shows that among the 101 patients, survivors (n = 87) had a lower mean NEWS2 score of 4.36 ± 2.698, while non-survivors (n=14) had a significantly higher mean score of 13.14 ± 1.406. This highlights the NEWS2 score's effectiveness in predicting patient outcomes, with higher scores associated with increased mortality risk and vice versa.

**Table 4:** Alive and death ratio status

Alive and death ratio status	Frequency (n)	Percent (%)	Mean ± std. deviation	Std. error mean
Alive	87	86.1	4.36 ± 2.698	0.289
Death	14	13.9	13.14 ± 1.406	0.376

**Figure 4:**
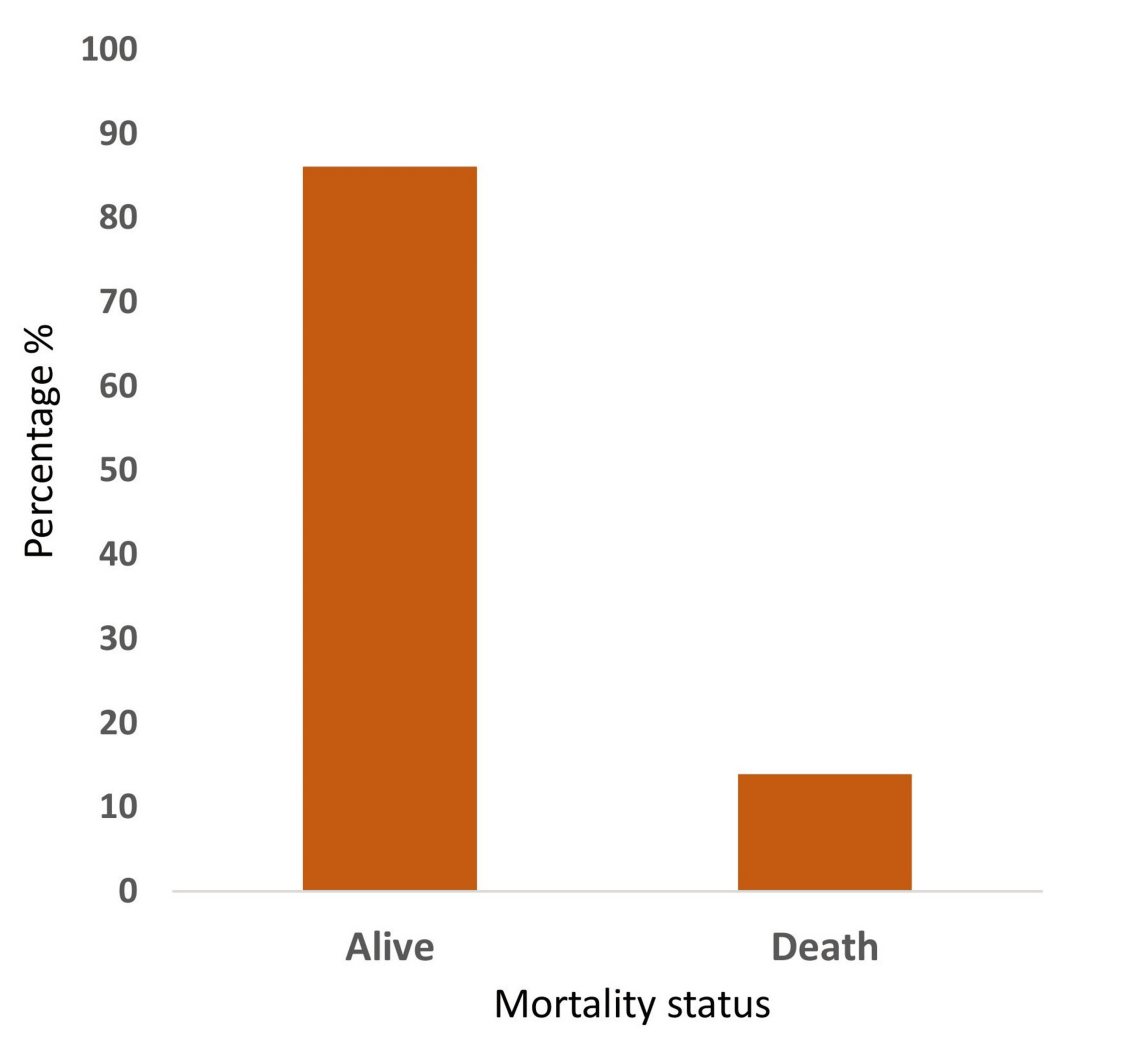
Alive and death ratio status

Figure 4 shows the ratio of the number of survivors and non-survivors among the population that participated in the study. Of the 101 patients, 86.1 % (n =87) survived and 13.9 % (n=14) died. 

## Discussion

This prospective study aimed to identify the effectiveness of NEWS2 in predicting the deterioration of patients admitted to emergency departments. Of the 101 participants included in the study, the data collection was conducted by recording the NEWS2 score before admission (baseline), and patients were followed up throughout their hospital stay, focusing on mortality progression and predictability.

Our study primarily indicated that NEWS2 showed strong predictive accuracy with respect to the mortality of patients admitted to the emergency department, highlighting the prospects of its clinical use. The statistical (t = 11.893, p < 0.05) and numerical difference in mean NEWS2 scores between survivors (4.36 ± 2.698) and non-survivors (13.14 ± 1.406) note their application as a valuable prognostic tool in acute care environments. The result complements the findings of Wibisono et al., where the study noted a high sensitivity and specificity of NEWS2 in the prediction of mortality of COVID-19 patients, affirming it to be a reliable indicator. Even though this study does focus on a specific condition, the findings are comparable and provide insights into the potential of NEWS2 in Indian healthcare [15].

Unlike other studies where NEWS2 was utilized to predict disease progression and mortality in specific conditions, like sepsis and COVID-19, our study stands novel without confining to a specific condition, thereby highlighting the generalizability of NEWS2's predictive accuracy in different clinical conditions [18-20]. We identified that the higher the NEWS2 score, the higher the mortality rate, as depicted in Table 3. Eighty-seven (86.1%) patients, who had a mean NEWS2 of 4.36 (SD ± 2.69), survived, while 14 (13.9%) patients, who had a mean NEWS2 of 13.13 (SD ± 1.406), died, as represented in Table 4 and Figure 3. Our study included study samples who were not confined to a specific condition, hence highlighting the generalizability of results.

In emergency settings, the observed mortality rate of 13.9% is notably lower than previous studies, such as the one by Hsieh et al. (42.92%), particularly those focusing on sepsis [21]. This difference could be attributed to our broader patient population, the effectiveness of early interventions guided by NEWS2, or specific characteristics of our healthcare setting. This difference could also be influenced by variations in the study duration.

There is an individual component analysis of each NEWS2 parameter that provides insights into which physiological parameters contribute most significantly to the overall score. Respiratory rate showed a relatively even distribution; however, a high proportion of patients scored 0 for systolic blood pressure (62.4%), which suggests that this parameter may be less discriminative in our population, possibly due to compensatory mechanisms in the early stages of deterioration. One notable finding is that the LOC showed a binary distribution (69.3% at 0, 30.7% at 3), while the pulse rate had the highest distribution at a score of 1 (53.5%), indicating signs of physiological stress. With respect to circulatory parameters, systolic blood pressure seemed a little deranged, with 62.4% of patients scoring 0. This finding notes that blood pressure can be considered a contributor to NEWS2 at earlier stages of illness. The high proportion of patients with mild tachycardia (53.5% scoring 1 for pulse rate) further supports the notion of early presentation or early stages of physiological stress.

NEWS2 score has more predictive value and diagnostic predictability with respect to other scoring/risk stratification tools. Verma et al. noted that NEWS2 is superior compared to qSOFA scores [20]. Similar findings were noted by Marosi et al., that the NEWS2 score, in comparison with qSOFA and SIRS criteria, has better accuracy in predicting mortality and sepsis risk within 72 hours [19]. Our study also highlights the predictive accuracy, thereby enabling better diagnosis and risk stratification of patients.

We recommend performing future studies that assess the effectiveness of NEWS2 scores in different settings, such as primary and secondary healthcare settings and rural clinics, to ensure NEWS2 can be applied across healthcare settings. Research should also aim to conduct randomized controlled trials (RCTs) to establish the causality between NEWS2 score and patient outcomes based on the different levels of intervention. With the increasing use of digital technology in healthcare, the possibility of integrating NEWS2 scoring with electronic health records (EHRs) can enhance timely interventions, generating real-time alerts under one system even when they are transferred to different departments. Further studies should explore the effectiveness of NEWS2 across different patient demographics, such as those with comorbidities, the elderly, or pediatric populations.

Strengths and limitations

Unlike studies that focus solely on the predictability of NEWS2 for specific conditions like COVID-19 or sepsis, our study utilized a population with multiple comorbidities, which allowed for broader generalization of the results. Additionally, our study primarily focused on a South Indian population and specifically prioritized identifying the distribution of individual NEWS2 parameters, an aspect not addressed by other studies.

However, our study had limitations, such as a small sample size, which could impact the generalizability of the results to larger populations. Furthermore, as a single-center study conducted in a tertiary care hospital in South India, the findings may not be universally applicable to all emergency departments or patient demographics, especially given the specific geographic context.

## Conclusions

Our findings demonstrate that higher NEWS2 scores at admission are strongly associated with mortality rates, validating NEWS2's effectiveness in identifying patients at risk of deterioration. This affirms its role as a critical tool for early intervention in emergency care.
